# Genome-wide identification and in silico analysis of GSTs reveals hormone mediated stress response in saffron

**DOI:** 10.3389/fpls.2025.1676384

**Published:** 2026-01-09

**Authors:** Muqaddas Bano, Xingnuo Li, Fang Liu, Ahmad Ali, Ejaz Hussain Siddiqi, Aidi Zhang, Xiujun Zhang

**Affiliations:** 1 School of Mathematics and Statistics, Wuhan University of Technology, Wuhan, China; 2 State Key Laboratory of Plant Diversity and Specialty Crops, Wuhan Botanical Garden, Chinese Academy of Sciences, Wuhan, China; 3 University of Chinese Academy of Sciences, Beijing, China; 4 National Key Laboratory of Crop Genetics Improvement, Huazhong Agricultural University, Wuhan, China; 5 Department of Botany, University of Gujrat, Gujrat, Pakistan

**Keywords:** genome-wide analysis, GSTs, saffron, secondary metabolite biosynthesis, stress response

## Abstract

Glutathione S-transferases (GSTs) are multifunctional enzymes in plants that facilitate stress management, detoxification of deleterious chemicals, and transport of secondary metabolites. This research performed a genome-wide examination of *Crocus sativus* (saffron) and discovered 29 GST genes, each possessing conserved N-terminal and C-terminal domains essential for their functionality. Phylogenetic study categorized these genes into subfamilies, including Tau, Phi, Theta, and Lambda, elucidating evolutionary tendencies unique to saffron. Structural research revealed many motifs and domains within the GST family, whereas chromosomal mapping demonstrated gene clustering, suggesting that gene duplication facilitated the growth of this gene family. Analysis of gene promoters identified regulatory regions that respond to hormones such as methyl jasmonate and abscisic acid, indicating the involvement of GSTs in stress reactions. Subcellular localization predictions indicated that the majority of GSTs are situated in the cytoplasm, with a few located in chloroplasts or vacuoles, highlighting their diverse functions. Structural modeling of a representative GST protein showed a two-subunit structure with distinct regions for binding and catalyzing reactions, validated by a high-quality model score and interactions with a test compound (S-hexylglutathione). This study provides a comprehensive analysis of the GST gene family in saffron, elucidating their structure, evolution, and activities. These insights establish a foundation for future research on the contributions of GSTs to stress tolerance and the synthesis of valuable compounds such as crocin and safranal, which may enhance saffron productivity.

## 1 Introduction

Glutathione S-transferases (GSTs; EC 2.5.1.18) are phase II metabolic isozymes common in fungi, mammals, and plants (Dixon et al., 2002). The first report of GSTs in plants dates back to 1970, when research revealed that GSTs extracted from maize participated in herbicide metabolism by facilitating the conjugation of synthetic chemicals with glutathione [GSH; γ-Glu–Cys–Gly] (Frear and Swanson, 1970). Plant GST proteins are globular dimers, typically consisting of two separate N- and C-terminal domains (Sylvestre-Gonon et al., 2019). The N-terminal domain comprises β-strands and α-helices in a thioredoxin-like fold, with a GSH-binding site (G-site) exhibiting high substrate specificity. The C-terminal domain is composed solely of α-helices, including an electrophilic substrate binding site (H-site) characterized by hydrophobic properties and extensive substrate specificity. This region accommodates many substrates and ligands (Dixon and Edwards, 2010; Dixon et al., 2002). Previous studies have classified plant GSTs categorize into 14 classes, including Phi, Zeta, Tau, Theta, Lambda, Ure2p, EF1Bγ, hemerythrin, Iota, Glutathionyl-hydroquinone reductases(GHRs) (Kitamura et al., 2004), Dehydroascorbate reductase(DHAR), Tetrachlorohydroquinone dehalogenase (TCHQD), Metaxin, and Microsomal prostaglandin E synthase type 2 (mPGES-2) (Shi et al., 2025). Among these, Tau, Phi, DHAR, and Lambda are plant-specific GST classes. Tau and phi GST classes are widespread in plants because of their vital functions in stress responses and detoxification activities, crucial for the plant adaptability to diverse environmental conditions. The *Arabidopsis thaliana* GST gene family consists of 57 members, categorized into nine classes, including 13 Phi and 28 Tau members (Dixon et al., 2009). Previous studies have also demonstrated the functional diversity among GST genes (Jain et al., 2010; Lan et al., 2009). Despite limited understanding, Iota class GSTs appear to function as oxidoreductases rather than conjugating GSTs, while hemerythrin facilitate the detoxification of heavy metals by promoting the conjugation of GSH with metal ions. Recent research has demonstrated the scavenging ability of members of Tau class against most hazardous reactive carbonyl species (RCs) (i.e. acrolein) and proposed their participation in defense mechanism against RCs (French et al., 2008). The functions of GST classes apart from Tau and Phi remain poorly understood. DHAR GSTs diminished the production of ascorbic acid within the ascorbate glutathione cycle to produce antioxidants (Moons, 2005). GSTs of the Zeta class exhibit isomerase activity and are involved in tyrosine catabolism (Edwards and Dixon, 2005). Theta class GSTs function as glutathione peroxidases, contributing to the metabolism of oxidative stress (Basantani and Srivastava, 2007). Lambda GSTs use flavonols as high-affinity ligands. Dixon and Edwards (Dixon and Edwards, 2010) elucidated a unique glutathione-dependent function of these enzymes in the recycling of oxidized quercetin. DHAR and Lambda GSTs exhibited action towards dehydroascorbic acid (DHA) (Liu et al., 2013).

GSTs primarily facilitate the conjugation of reduced tripeptide glutathione (GSH) to diverse reactive electrophiles. GST proteins are crucial for the appropriate growth and physiological functioning of plants. They are crucial in stress response (Csiszár et al., 2014; Roxas et al., 2000) and participate in the breakdown of insecticides and herbicides (Smith et al., 2003). GSTs exhibit responsiveness to plant hormones, including auxins, ethylene, salicylic acid, abscisic acid, and jasmonic acid (Marrs, 1996; Moons, 2005). Based on current advances in plant proteomics, genomics, and transcriptomics studies, Chronopoulou et al. (2017) examined the functions of GSTs. Apart from their catalytic activity, GSTs bind to flavonoids and anthocyanins, therefore acting as non-catalytic proteins by moving them from the cytoplasm into the central vacuole (Kitamura et al., 2004). Additionally via binding to porphyrinogens (Dixon et al., 2008) and oxylipins (Dixon et al., 2009), GSTs protect plant cells from the oxidative stress (Sappl et al., 2009; Shen et al., 2015). Plant GSTs are also essential in responses to biotic and abiotic stresses such as pathogen attacks, heavy metals (Gunning et al., 2014), dehydration (Xu et al., 2015) and salinity (Sharma et al., 2014), as well as salicylic acid signaling (Liu et al., 2013). The precise methods remain unidentified. Furthermore, GSTs may play a role in the transport and the metabolism of secondary metabolites, including anthocyanins, flavonoids, and porphyrins (Dixon and Edwards, 2010).

*Crocus sativus* (saffron), a valuable horticultural crop, is esteemed for its stigmas, which produce the most costly spice globally. The cultivation is impeded by abiotic factors (e.g., drought, salinity) and biotic stresses (e.g., fungal diseases), which diminish production and quality. GSTs are essential enzymes in plant stress responses, facilitating the detoxification of reactive oxygen species and modulating hormone signaling. Recent genome-wide analysis has identified 53 GST genes in Arabidopsis (Sappl et al., 2009), 79 in rice (Jain et al., 2010; Soranzo et al., 2004), 25 in soybean, 42 in maize (McGonigle et al., 2000), 90 in tomato (Islam et al., 2017), 85 in pepper (Islam et al., 2019), 32 in pumpkin (Abdul Kayum et al., 2018), 23 in sweet orange (Licciardello et al., 2014), 90 in potato (Islam et al., 2018), and 20 in *Dracaena cambodiana* (Zhu et al., 2016). Despite the identification of GSTs as contributors to plant stress tolerance and detoxification, a comprehensive analysis of the GST gene family in saffron remains unavailable. The absence of genetic and functional insights limits our understanding of the roles saffron GSTs play in stress adaption and overall plant resilience. Understanding GST operations may facilitate improvements in saffron production and quality among environmental challenges. This study conducted a genome-wide annotation and phylogenetic analysis of the GST gene family in *C. sativus*. Using the computational tools such as BLAST (Ye et al., 2006), TBtools (Chen et al., 2020), and Python scripts, we identified and characterized GST genes, examined their motif structures, and assessed their physicochemical characteristics. Comparative analysis with *Arabidopsis thaliana* and other plant species revealed lineage-specific gene divergence and evolutionary trends. Promoter region analysis and phylogenetic tree construction provided deeper insights into the regulatory and functional diversity of GST genes. Despite the economic importance of saffron, a comprehensive genome-wide investigation of GSTs is lacking, which constrains our understanding of its mechanisms for stress tolerance.

## 2 Materials and methods

### 2.1 Identification of GST genes

We utilized a diverse collection of the GST protein sequences from various plant species, such as *Arabidopsis thaliana* (Arabidopsis), *Sedum alfredii* (Stone crop), *Pyrus pyrifolia* (pear), *Elaeis guineensis* (Oil palm), *Populus ussuriensis* (Poplar), *Hordeum vulgare* (Barley), *Pisum sativum* (Pea), *Noccaea caerulescens* (Pennycress), *Caragana korshinskii* (Pea shrub), *Solanum elaeagnifolium* (Nightshade), and *Glycine max* (Soybean), to conduct BLASTP searches against the annotated protein sequences of the *Crocus sativus* genome dataset, sourced from Figshare (DOI: 10.2984/m9.figshare.21988667.v1). To ensure the identification of a diverse range of homologous sequences, BLASTP searches were performed with an e-value threshold of ≤ 1e-20 and a maximum of 50 results per query. This conservative cutoff is widely used in plant GST gene family studies (Islam et al., 2017; Jain et al., 2010). Targeted searches in the Pfam (Bateman et al., 2004) and SMART databases (Letunic et al., 2004) were used to confirm the results, which showed that the sequences included conserved domains unique to GST. The genome of *C. sativus* included 29 distinct GST genes after eliminating duplication and non-GST sequences. To remove any possibility of duplicates, we used CD-HIT with a 100% sequence identity cutoff to keep only unique nucleotide sequences. The dataset was further refined by using ClustalW for multiple sequence alignment cleaning and manual curation in order to exclude any mismatched or unclear sequences. We organized the genes into a FASTA (Pearson, 1994) file so that we could do structural investigations, evolutionary connection analyses, and motif identifications and other analyses. A strong basis for future functional and evolutionary investigations is provided by this curated list of *C. sativus* GST genes. The *C. sativus* GST gene pool has been successfully increased by this method, which is encouraging for next functional and evolutionary studies.

### 2.2 Phylogenetic analysis

Protein sequences of 29 C*. sativus* glutathione S-transferases (*CsGST1-29*) were acquired from a protein dataset available on Figshare (DOI: 10.2984/m9.figshare.21988667.v1). Six *Arabidopsis thaliana* glutathione S-transferases *(AtGST1-6)* were obtained from the TAIR (https://www.arabidopsis.org/). To augment taxonomic diversity, 27 supplementary GST sequences from diverse plant species, encompassing monocots (*Zea mays [ZmGST1]*, *Oryza sativa [OsGST1–2]*, *Lolium perenne [LpGST1]*), eudicots (*Glycine max [GmGST1-2]*, *Solanum lycopersicum [SlGST1-2]*), and gymnosperms (*Pinus brutia [PbGST1]*), were obtained from the NCBI (https://www.ncbi.nlm.nih.gov/) Protein Database. Sequence identifiers for all analyzed GSTs are available in **Supplementary Table S1**, **Sheet 3**. Sequences were aligned with ClustalW (https://www.genome.jp/tools-bin/clustalw) to provide multiple sequence alignment. A phylogenetic tree was generated using RAxML v8.2.12 with the PROTGAMMABLOSUM62 model, using 1000 bootstrap iterations and approximate likelihood ratio test (aLRT) support to evaluate node confidence. *Sedum alfredii (SaGST1)*, sourced from the NCBI Protein Database, served as the outgroup owing to its evolutionary divergence from monocot and eudicot GSTs, hence providing a robust tree topology. GSTs were classified into Phi, Tau, Theta, and Lambda sub classes according to Pfam domain profiles (e.g., PF02798 for the N-terminal, PF00043 for the C-terminal), and sequence similarity evaluated through BLASTp against the NCBI Protein Database (threshold: >29% identity for the same subclass). Subclass assignments were validated by analyzing active-site residues in the alignment using Jalview, with Tau GSTs distinguished by a conserved serine and Phi GSTs identified by hydrophobic residues in the substrate-binding site.

### 2.3 Motif analysis

The MEME (https://meme-suite.org/meme/) was used to find conserved motifs among GST proteins of *C. sativus*. Using default settings for motif width and significance thresholds, the protein sequences found in the previous phase were analyzed under criteria allowing the detection of up to 10 motifs. By cross-referencing these motifs with domain annotations, their functional importance was evaluated. The functional domains of GST proteins were confirmed using the Pfam (http://pfam.xfam.org/) and SMART databases, which identified characteristic GST domains, including C-terminal alpha-helical domain and the N-terminal thioredoxin-like domain. The processed data were systematically organized to facilitate visualization and further interpretation in the results stage. These analysis provided insights into the conserved structural elements and functional roles of GST.

### 2.4 Domain validation

Domain validation using the Pfam database was performed to confirm the identification of GST genes in *C. sativus*. Conserved domains characteristic of the GST family were identified, including the alpha-helical C-terminal domain (GST_C) and the thioredoxin-like N-terminal domain (GST_N). Reflecting the diversity of GST proteins in *C. sativus*, variations of these domains, such as GST_N_2, and GST_N_3, as well as GST_C_2, GST_C_3, and GST_C_5 were also found. These domain variations highlight the structural diversity of the GST gene family in *C. sativus*. Only genes containing conserved GST-specific domains were included in the final dataset for further phylogenetic and functional analysis.

### 2.5 Ka/Ks analysis of GST gene sequences

Synonymous (Ks) and non-synonymous (Ka) substitution rates were calculated to evaluate the selective pressures acting on the GST gene family in *C. sativus.* To ensure high-quality alignment for subsequent analysis, coding sequences (CDS) of the identified GST genes were aligned using the ClustalW method in the MEGA program. Subsequently, a neighbor-joining phylogenetic tree was constructed from the aligned sequences to establish an evolutionary framework and guide pairwise comparisons. The Codon-Based Test of Selection in MEGA computed pairwise Ka/Ks values. The Ka/Ks ratio of each gene pair provided insight into the type of selection acting on the sequences. Ratios greater than 1 suggested positive selection, adaptation to specific functional or environmental pressures. Ratios less than 1 suggested purifying selection, which works to preserve gene functionality by removing harmful mutations. A ratio equal to 1 indicated neutral evolution. Finally, the pairwise distance data, including the Ka/Ks ratios, were saved in an Excel file for further analysis. This approach facilitated the assessment of the evolutionary processes influencing the GST gene family in *C. sativus*.

### 2.6 Chromosomal distribution and synteny analysis

The *C. sativus* genome annotation provided the chromosomal locations of the 29 *CsGST* genes, which were then visualized using TBtools-II. MCScanX with default settings was used to conduct intraspecific and interspecific synteny studies. Collinear blocks with a minimum of five gene pairs were kept, and TBtools-II (Chen et al., 2023) Advanced circos and multiple synteny plot were used to visualize the data.

### 2.7 Predicting subcellular localization

WolfPsort (https://wolfpsort.hgc.jp/), a computational technique designed to predict protein localization based on amino acid sequences, was used to project the subcellular localization of GST proteins in *C. sativus*. The amino acid sequences of all 29 identified GST genes were submitted to the WolfPsort server. The tool analyzed the sequences and assigned a score for each protein across various cellular compartments, including the cytoplasm, chloroplast, nucleus, vacuole, mitochondria, cytoskeleton, extracellular space, and others. These scores represent the likelihood of a protein localizing to a particular compartment. The compartment with the highest score was considered the primary localization site for each protein. If significant scores were observed, secondary and tertiary localization predictions were also considered to provide a comprehensive view of the subcellular location of each GST protein. The resulting data were compiled for further display and analysis.

### 2.8 Physicochemical property analysis

The structural and functional characteristics of the identified GST proteins in *C. sativus* were analyzed through physicochemical property assessments. The ExPASy (https://www.expasy.org/) was used to compute key parameters, including molecular weight (kDa), isoelectric point (pI), and hydrophobicity (GRAVY score). FASTA-format protein sequences were input into ProtParam, and the results for each sequence were recorded. The GST proteins were classified based on their physicochemical profiles, including their acidic or basic nature (based on pI) and their hydrophilic or hydrophobic tendencies (based on the GRAVY score). This analysis is crucial for predicting the biochemical functions of GST proteins and selecting candidates for further functional studies. By providing insights into the stability, solubility, and functional significance of these proteins, this analysis lays the groundwork for experimental validation and comparative studies.

### 2.9 Examination of cis-regulatory elements

Promoter sequences of 29 C*. sativus* glutathione S-transferase (GST) genes (*CsGST1* to *CsGST29*) were obtained using TBtools v1.098 from the *C. sativus* genome assembly and annotation (source: DOI:10.2984/m9.figshare.21988667.v1). A 2000 bp segment upstream of the designated transcription start site (TSS) was retrieved for each gene, since this length is conventional for identifying cis-regulatory elements in plant promoters. To rectify any mistakes in TSS annotations arising from computational predictions in a non-model plant genome, sequences were cross-validated against the translational start site (ATG) to confirm the presence of regulatory regions. The recovered sequences were examined for cis-acting regulatory elements using the PlantCARE (http://bioinformatics.psb.ugent.be/webtools/plantcare/html/) database, which forecasts motifs based on empirically confirmed data from plant species. The analysis concentrated on hormone-related cis-elements, specifically methyl jasmonate (MeJA)-responsive (CGTCA-motif, TGACG-motif), abscisic acid (ABA)-responsive (ABRE), and gibberellin-responsive (GARE-motif, P-box, TATC-box) elements, owing to their significance in stress-mediated GST regulation. Additionally, it identified other hormone-responsive (e.g., salicylic acid, auxin, ethylene), stress-responsive (e.g., MYB binding sites, anaerobic induction), light-responsive (e.g., G-box, Box 4), and regulatory motifs (e.g., cell cycle, conserved DNA modules). The distribution of cis-elements was shown using TBtools, producing promoter maps, and their frequencies were categorized (**Supplementary Table S7**).

### 2.10 Gene ontology enrichment analysis

To clarify the functions of differentially expressed *CsGST* genes (FDR < 0.05), we conducted Gene Ontology (GO) (https://geneontology.org/) enrichment analysis using annotations sourced from the eggNOG-mapper v2 web server (http://eggnog-mapper.embl.de/). The target gene set included 18 *CsGST* genes *(CsGST1, CsGST2, CsGST3, CsGST8, CsGST9, CsGST12, CsGST13, CsGST14, CsGST15, CsGST16, CsGST17, CsGST19, CsGST20, CsGST21, CsGST24, CsGST25, CsGST26, CsGST27)* recognized as highly differentially expressed in comparisons of stigma or tissue. Gene Ontology annotations for these genes were obtained from the eggNOG-mapper output (file: out.emapper.annotations.GO.txt). We used GO annotations for cucumber (*Cucumis sativus*), as a background gene set, sourced from UniProt, to ensure relevance to plant stress responses. The Gene Ontology enrichment study was performed with the GOATOOLS Python package (version 1.2.3). The GO ontology file (go-basic.obo) was obtained from (http://purl.obolibrary.org/obo/go/go-basic.obo), and the GO Directed Acyclic Graph (DAG) was generated using GODag from GOATOOLS. Gene-to-GO word relationships were established for both target and background genes by analyzing the annotation files using pandas. To concentrate on hormone-mediated stress responses, pertinent Gene Ontology (GO) terms were filtered for keywords associated with hormones and stress (hormone, abscisic acid, cytokinin, gibberellin, ethylene, jasmonic acid, salicylic acid, auxin, stress response, cellular response to stress) utilizing pandas and numpy. The filtered findings were visualized using Seaborn and Matplotlib, emphasizing hormone-related Gene Ontology words in a unique dark orange hue, contrasted with other terms in light steel blue.

### 2.11 Structural and interaction analysis of *CsGST* proteins

Homology modelling of the 29 C*. sativus* GST proteins (*CsGST1-CsGST29*) was conducted via SWISS-MODEL (https://swissmodel.expasy.org/) to forecast their three-dimensional structures, emphasizing the N-terminal and C-terminal regions essential for catalytic activity. Templates were chosen from the Protein Data Bank (PDB) according to sequence identity (≥30%) and structural resolution (<2.5 Å). The ProMod3 engine enabled model generation and refining, with model quality assessed using the QMEAN scoring tool, producing values ranging from -0.93 *(CsGST1, CsGST14)* to -0.86 *(CsGST7, CsGST9)*, signifying strong structural reliability. AlphaFold was utilized to forecast secondary structural components, namely for the N-terminal (residues 10-91) and C-terminal (residues 96-221) domains, and was cross-validated using PSIPRED predictions for α-helices and β-sheets. PyMOL rendered the models, coloring the N-terminal domain cyan and the C-terminal domain orange to differentiate their structures. Protein-protein interaction (PPI) networks were established utilizing the STRING database (https://string-db.org/) confidence threshold >0.4 and visualized with Cytoscape (v3.9.1) to clarify functional relationships.

### 2.12 RNA-seq analysis

**Figure 1:**
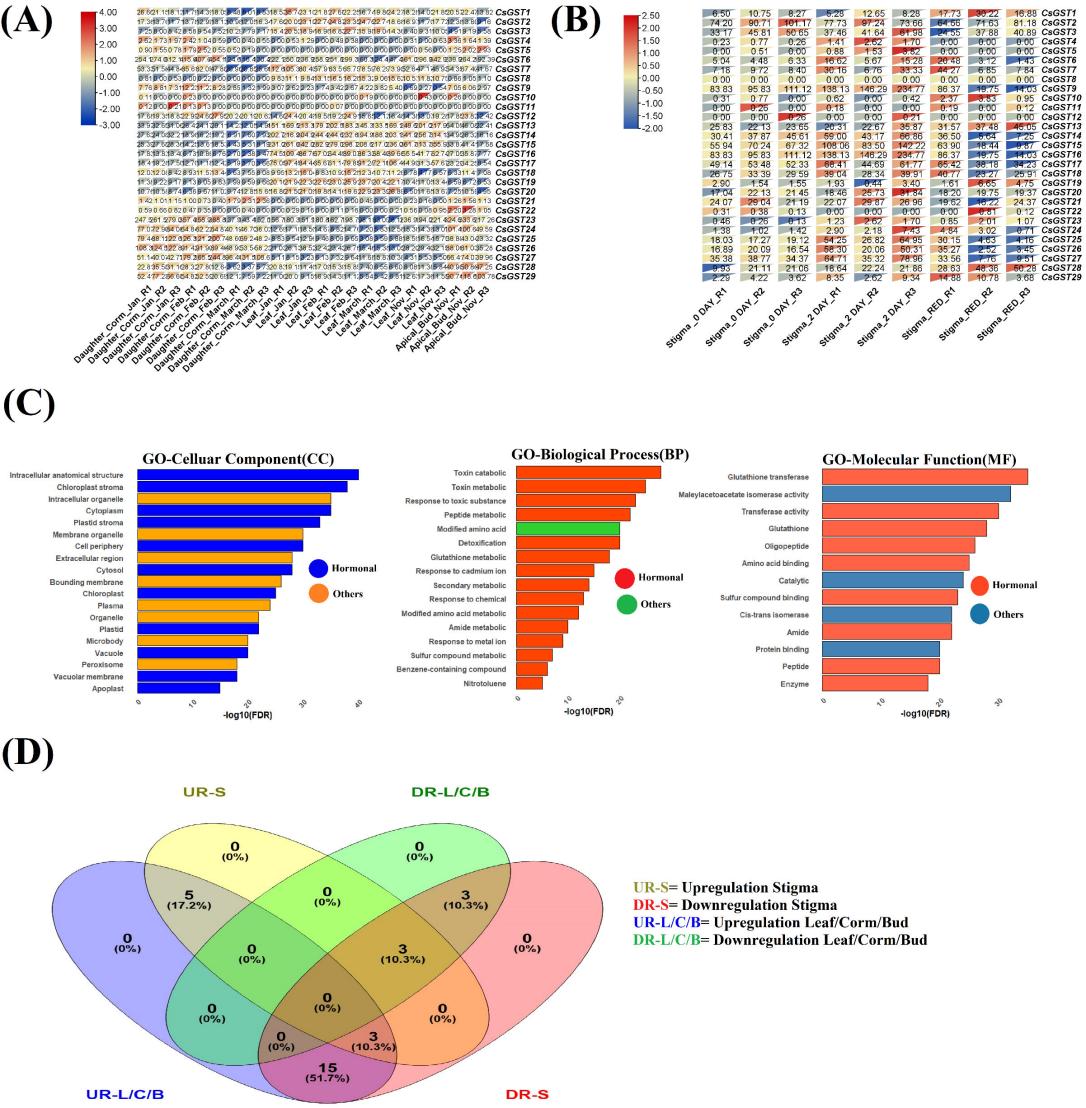
Comprehensive investigation of gene expression and regulatory enrichment in saffron tissues: **(A)** A heatmap illustrating the normalized expression levels of saffron genes across diverse tissue samples. Rows denote specific genes, whereas columns signify various tissue types. The color scale extends from blue (indicating low expression) to red (indicating high expression). **(B)** Temporal expression heatmap of stigma-specific genes across developing days post-treatment. Expression levels are normalized, emphasizing significant transcriptional changes throughout stigma. **(C)** Gene Ontology (GO) enrichment analysis demonstrating considerably enriched terms across three categories: Biological Process (BP), Molecular Function (MF), and Cellular Component (CC). The bars denote -log10(FDR) values, with color-coded panels distinguishing hormonal functions from non-hormonal functions. **(D)** A Venn diagram illustrating the intersection of differentially expressed genes (DEGs) between stigma-specific regulation (UR-S, DR-S) and vegetative tissues (UR-L/C/B, DR-L/C/B). The figures and percentages denote the DEG distribution among distinct and overlapping groups.

We used two publicly available RNA-seq datasets from the NCBI Sequence Read Archive (SRA): PRJNA400472 and PRJNA976833, to investigate the expression patterns of GST genes in *C. sativus* and their hormonal modulation by methyl jasmonate (MeJA), abscisic acid (ABA), and gibberellic acid (GA). The PRJNA400472 dataset contains transcriptomic data from stigma tissues at three developmental stages (0 DAY, 2 DAY, RED(mature stage)). The PRJNA976833 dataset comprises transcriptomic profiles from various tissues, including daughter corm, leaf, and apical bud, collected at multiple time points (January, February, March, November), with three biological replicates per condition, enabling the investigation of tissue-specific expression during bud dormancy and stress responses in November. Expression data were reported in Transcripts Per Million (TPM), which adjusts for gene length and sequencing depth. For differential expression analysis, TPM data were averaged across replicates for each condition, and a pseudocount of 0.0095 was included to address zero values. Log2 fold change (logFC) was computed as log_2_((TPM_condition1 + 0.0095)/(TPM_condition2 + 0.0095)), contrasting Stigma_RED with Stigma_0_DAY (PRJNA400472) for stigma development and Apical_Bud_Nov with Leaf_Nov (PRJNA976833) for tissue-specific responses. Because many plant GST genes exhibit low basal expression and biologically relevant responses often manifest as modest fold changes, differential expression was primarily determined using a false discovery rate (FDR) < 0.05 as the significance filter. Plant GST family studies typically address physiologically relevant genes with smaller fold-changes (0.1 < |log_2_FC| < 1) when FDR < 0.05 and expression trends are consistent across replicates, despite the fact that large-scale DEG lists usually utilize a |log_2_FC| ≥ 1 cutoff. Log_2_ fold-change (logFC) values were interpreted in the context of consistent directional trends across replicates, a common and accepted practice in GST family expression studies (Sappl et al., 2009). Statistical significance was assessed using DESeq2, which applies median-of-ratios normalization on raw counts and a negative binomial model to obtain p-values and FDR. Genes with FDR < 0.05 were classified into four categories: upregulated in stigma (UR-S), downregulated in stigma (DR-S), upregulated in leaf/corm/bud (UR-L/C/B), and downregulated in leaf/corm/bud (DR-L/C/B), as illustrated in the Venn diagram (**Figure 1**). In the comparison between Apical_Bud_Nov and Leaf_Nov, CsGST14 exhibited the highest expression difference among the 29 GST genes (logFC = 3.2), indicating strong tissue-specific upregulation in the bud (UR-L/C/B, **Figure 1**). The promoter regions of these GST genes were analyzed for MeJA-, ABA-, and GA-responsive elements to investigate hormone-mediated regulation, and their distribution among the four expression groups was shown using a Venn diagram. Heatmaps generated in TBtools demonstrated expression patterns, showcasing gene expression across several tissues under different situations (PRJNA976833) and in stigma tissue on Days R1, R2, and R3 during stress therapy (PRJNA400472).

## 3 Results

### 3.1 Discovery of GST genes in *C. sativus*

A comprehensive genome-wide study identified 29 GST genes in *C. sativus*. These genes are distributed across the genome and annotated based on the presence of conserved GST-specific domains, such as the C-terminal alpha-helical domain and the thioredoxin-like N-terminal domain. The 29 *CsGST* genes were named *CsGST1* to *CsGST29* sequentially according to their physical position from the top to the bottom of chromosomes 1 through 8 (i.e., from the short-arm telomere to the long-arm telomere on each chromosome). Comprehensive genomics data for the identified *C. sativus* GST genes are provided in **Supplementary Table S1**, which includes key information such as gene ID, gene name, chromosome, strand orientation (start and end), and amino acid count. This dataset offers a detailed overview of the genes, highlighting their genomic locations, structural features, and potential functions, serving as a foundational resource for further research on the GST gene family in *C. sativus*.

### 3.2 Phylogenetic analysis of GST genes

**Figure 2:**
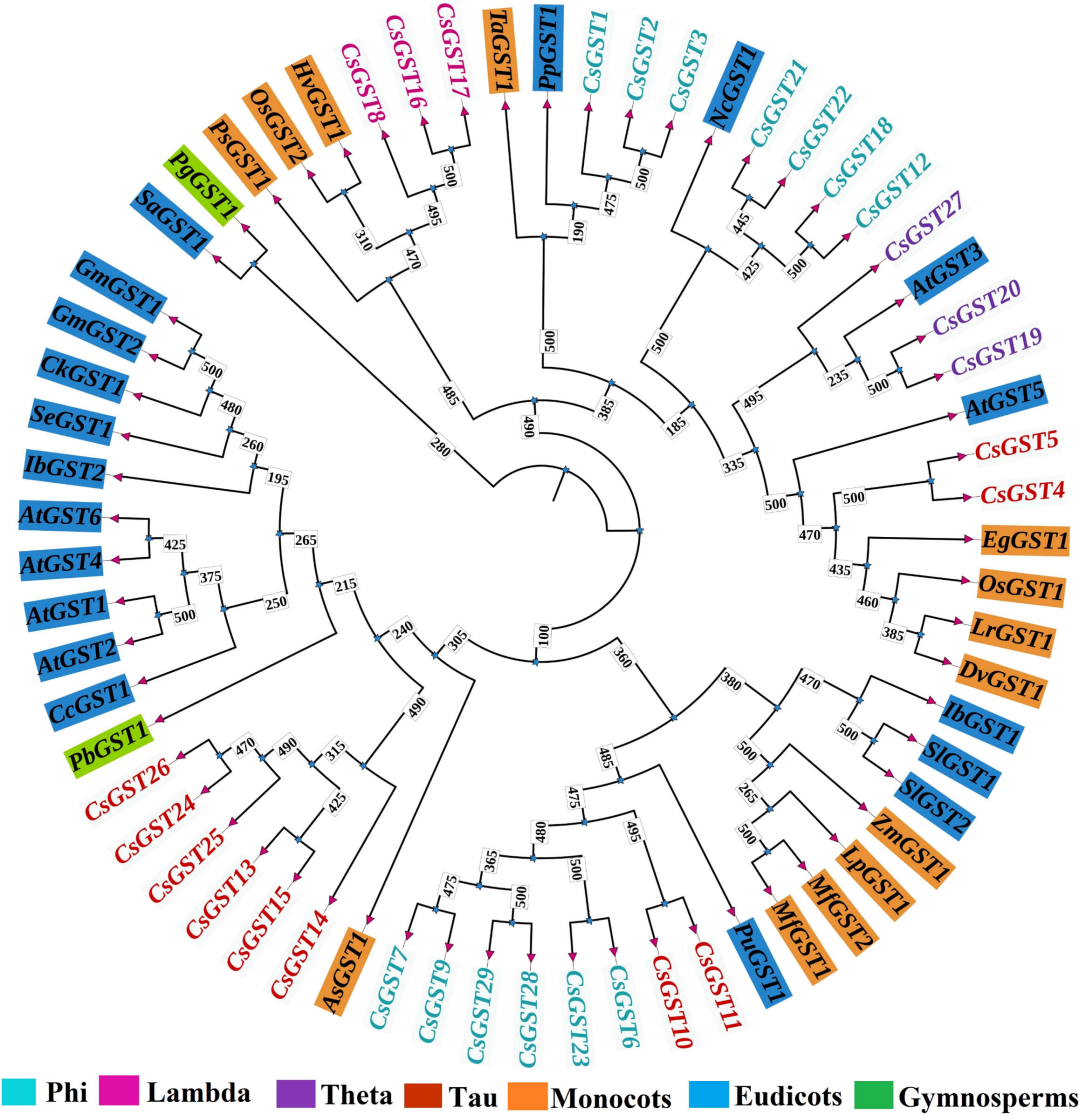
The phylogenetic tree showing the relationships among GST protein sequences from various species.

The RAxML phylogenetic tree, generated using 1000 bootstrap replicates and aLRT support, categorized *C. sativus* GSTs (*CsGST1-29*), *Arabidopsis thaliana* GSTs (*AtGST1-6*), and 27 supplementary plant GSTs from the NCBI Protein Database (**Supplementary Table S1**, Sheet 3) into separate clades aligned with GST subclasses (**Figure 2**). *CsGST1-3*, *6-9, 12, 18, 21-23, 28–29* were classified as Phi GSTs, establishing a strong clade with reference Phi GSTs, such as *ZmGST1 (Zea mays)*, *PpGST1 (Pyrus pyrifolia)*, and *TaGST1 (Triticum aestivum)*, with 95-99% bootstrap and aLRT support. These sequences had robust N-terminal (PF02798, e.g., 3.4e-17 for *CsGST3*) and C-terminal (PF13410, e.g., 1.5e-06 for *CsGST2*) domains, indicative of Phi GSTs. *CsGST4-5, 10-11, 13-15, 24–26* were categorized within the Tau subclass, clustering with *AtGST5 (Arabidopsis thaliana)* and *GmGST1 (Glycine max)* with 98-100% support, distinguished by significant PF00043 domains (e.g., 4.6e-17 for *CsGST4*). *CsGST19–20* and *CsGST27* were classified as Theta GSTs, aligning with *AtGST1 (AtGST3)* with 98-100% support, exhibiting weaker C-terminal domains (PF13410, e.g., 0.00085 for *CsGST19-20*). *CsGST8* and *CsGST16–17* were categorized as Lambda GSTs, exhibiting divergent domains (e.g., PF16865, 8.2e-07 for *CsGST8*; CLIC-like_N, PF22441) and clustering in proximity to *SaGST1* (tentatively classed as Lambda) with 90-95% support. *CsGST12* had a very negligible branch length, originally indicating possible duplication with *CsGST3* (Phi); nonetheless, it was kept as a Phi GST due to its unique sequence and domain profile (PF13410, 1.2e-13). The phylogenetic tree, anchored by *SaGST1*, emphasized monocot-enriched Phi clades (e.g., *CsGST1–3* with *ZmGST1*) and eudicot enriched Tau clades (e.g., *CsGST4–5* with *GmGST1*), highlighting lineage-specific GST diversification in *C. sativus*.

### 3.3 Motif and domain analysis conservation in GST genes

**Figure 3:**
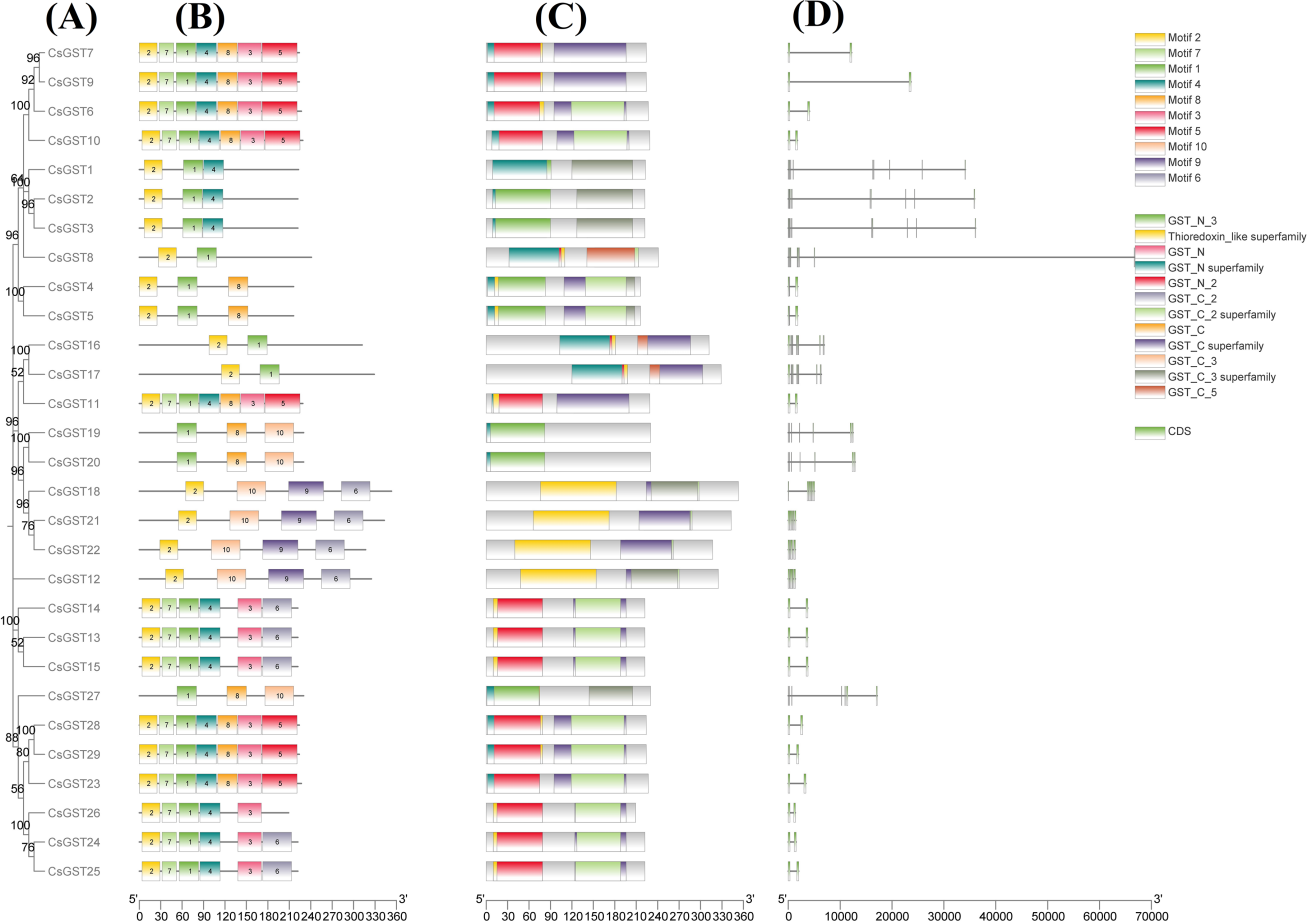
Phylogenetic connections, conserved motifs, domain patterns, and gene structures of *C. sativus* glutathione S-transferases. **(A)** Phylogenetic tree was created using the full protein sequences of *C. sativus* GSTs **(B)** Display of motifs identified in *CsGSTs* proteins. **(C)** Display of conserved domains identified in *CsGST* proteins. **(D)** Gene architecture of *CsGST*.

A detailed investigation of the GST genes in *C. sativus* revealed conserved sequence patterns critical for their structural and functional roles. Using the MEME Suite, ten distinct motifs were identified, and their positions along with corresponding Pfam values were recorded. These motifs, crucial for maintaining GST-specific enzymatic activity and binding properties, highlight conserved regions common across GST family members. The GST genes of *C. sativus* were classified according to their domain makeup, as outlined in **Supplementary Table S2**, with evolutionary connections shown in **Figure 2**. **Supplementary Table S3** presents the sequences, logo and width of each motif. Motif and domain analysis of the identified *Crocus sativus* GST genes revealed several conserved domains unique to the GST family. Consistent with canonical GST activity and structural stability, the N-terminal GST domains (e.g., GST_N, GST_N_2, GST_N_3) and C-terminal GST domains (e.g., GST_C, GST_C_2, GST_C_3, GST_C_5) were predominant throughout the sequences. **Figure 3** focuses on motif and domain structure. It depicts the genomics architecture of various gene sequences, including *CsGST* and GST family members. The evolutionary connections among different isoforms are represented in a tree-like structure, showing their association with specific motifs or protein domains. These motifs are mapped along the gene sequences, with each bar representing a different domain within the sequences. The image on the right shows additional structural annotations, including coding sequences (CDS) with conserved motifs and potential functional regions across the isoforms. The variability in motif distribution observed in this study may reflect functional specialization or evolutionary divergence within the gene family.

### 3.4 Ka/Ks analysis of GST gene sequences

To clarify the evolutionary and functional roles of GST genes in *C. sativus*, we performed an extensive investigation of their evolutionary selection, chromosomal distribution, subcellular localization, and physicochemical properties. The Ka/Ks analysis of GST gene pairs suggested that all pairings are subject to purifying selection, with Ka/Ks ratios between 0.0596 and 0.4868 (**Supplementary Table S4**). The *CsGST2-CsGST3* pair demonstrated the most pronounced purifying selection (Ka/Ks = 0.0596), indicating rigorous evolutionary pressures to maintain essential enzymatic functions, whereas the *CsGST12-CsGST18* pair, with the highest ratio (0.4868), suggests somewhat relaxed constraints, potentially facilitating functional divergence within conserved roles. Additional significant pairs, including *CsGST4-CsGST5* (Ka/Ks = 0.1180) and *CsGST28-CsGST29* (Ka/Ks = 0.0749), further emphasize strong purifying selection. The findings suggest that the GST gene family is evolutionary conserved, possibly preserving functional stability to facilitate saffron’s response to environmental challenges common in its Mediterranean and semi-arid habitats. GSTs specifically facilitate the conjugation of glutathione to xenobiotics and reactive oxygen species (ROS), thereby detoxifying toxic substances and conserving cellular components, which is essential for preserving the stigma during the formation of important apocarotenoids such as crocin and safranal.

### 3.5 GST genes chromosomal distribution and synteny analysis

**Figure 4:**
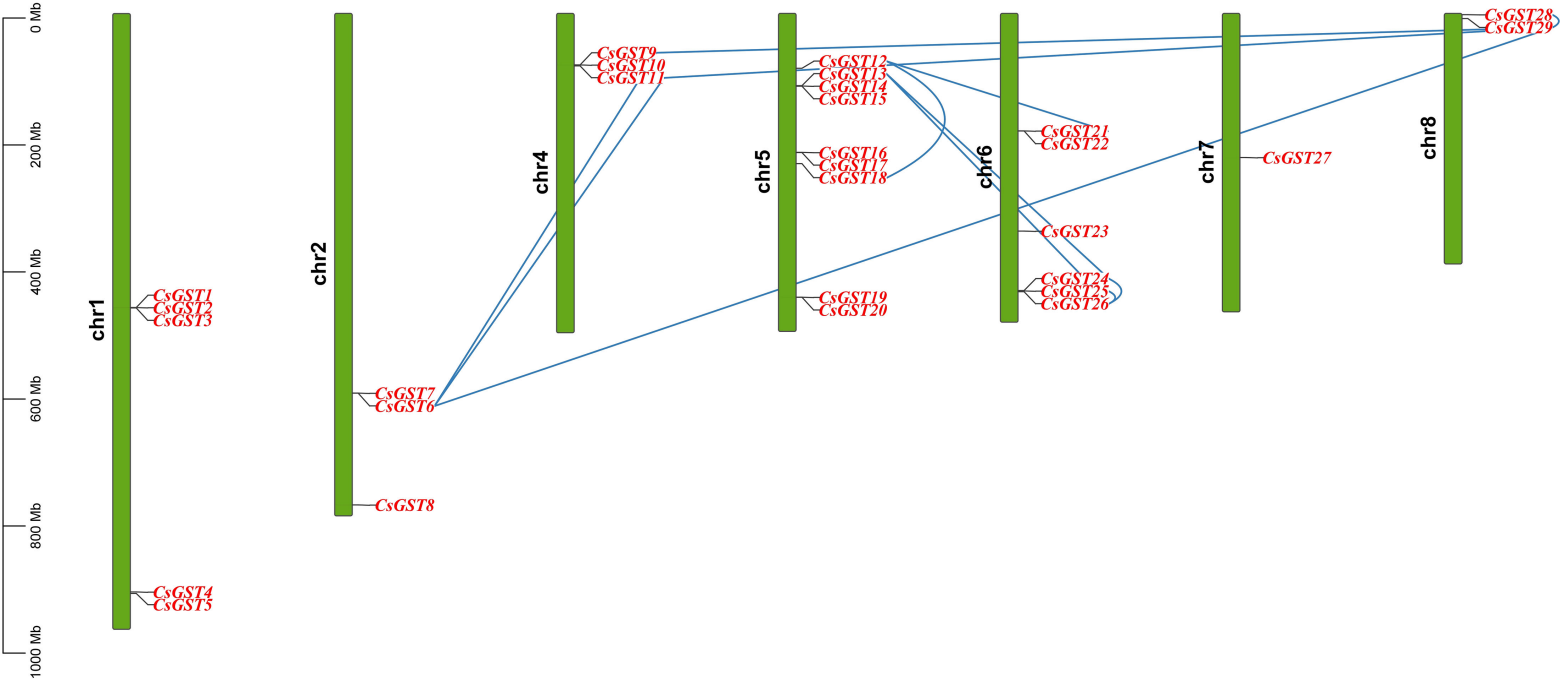
Genomic localization of GST genes in *C. sativus*. The 29 glutathione S-transferase (GST) genes are distributed over eight chromosomes (chr1 to chr8) as vertical bars, with significant clustering observed on chr1, chr5, and chr6, suggesting probable gene duplication and functional specialization influenced by triploidy. Blue lines represent syntenic relationships between gene pairs.

**Figure 5:**
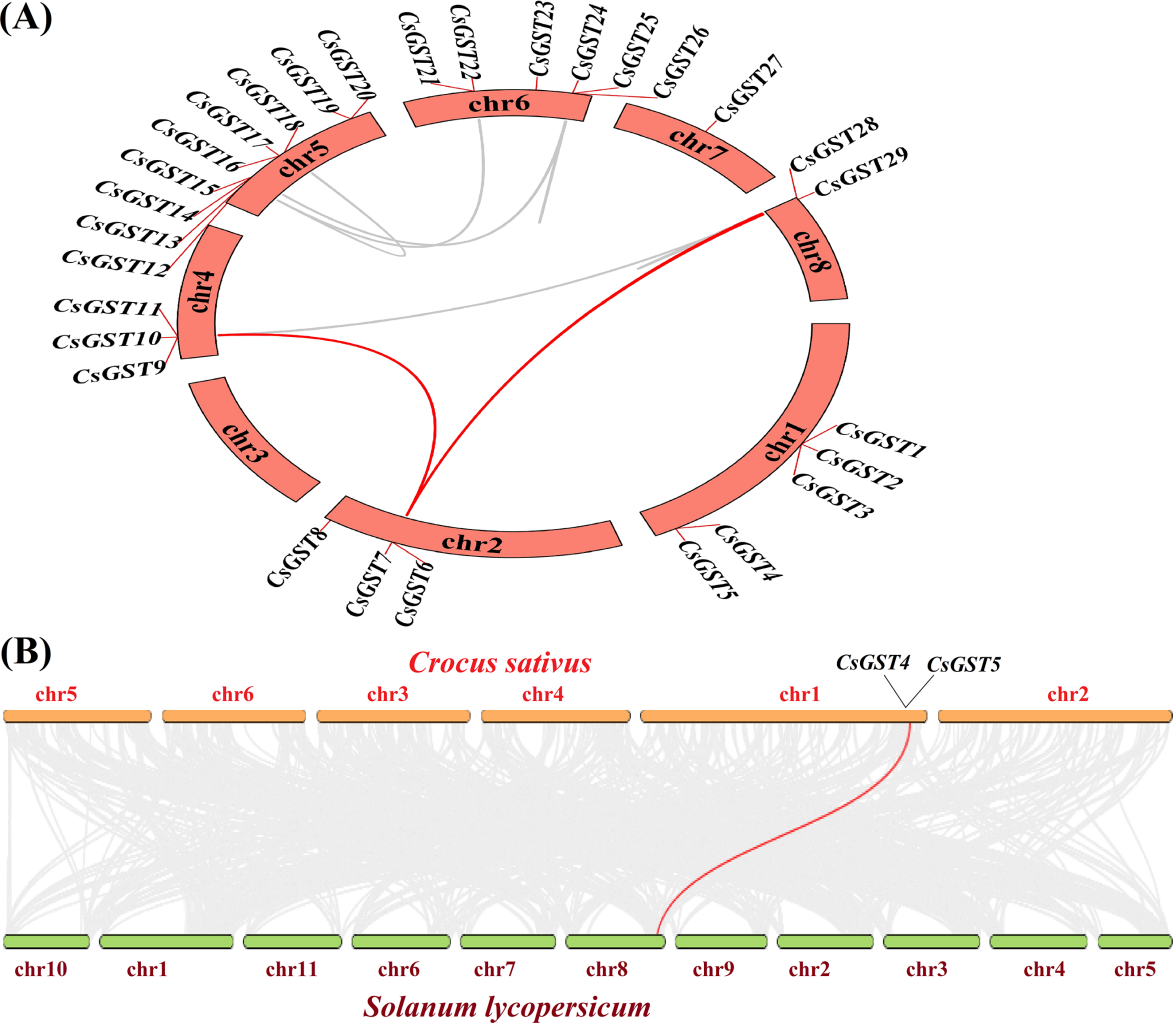
Synteny analysis. **(A)***CsGST* gene intraspecific synteny plot in the genome of *C. sativus*. The arrangement of the eight chromosomes is circular. All collinear blocks found in the saffron genome are shown by grey lines in the background, whereas syntenic *CsGST* gene pairs are highlighted by red curving lines. Twelve collinear pairings were found, suggesting segmental duplication as the primary route of growth. **(B)***C. sativus* (top) and *Solanum lycopersicum* (bottom) exhibit interspecific synteny. The evolutionary conservation of the GST family spanning monocots and eudicots is shown by the red line that link 14 orthologous GST gene pairs.

The 29 *CsGST* genes were distributed across eight chromosomes of *C. sativus*, with the greatest densities on chr1, chr5, and chr6, whereas chr3 had no GST genes at all (**Figure 4**). Twelve collinear *CsGST* gene pairs that were found within segmental duplication blocks without any indication of tandem duplications by intraspecific synteny analysis (**Figure 5A**). Major syntenic areas were identified between chr1-chr5, chr1-chr6, chr2-chr6, and chr5-chr6, consistent with documented whole-genome triplication occurrences in the *Crocus* lineage. This suggests that the expansion of the *CsGST* family has been primarily driven by segmental duplication rather than tandem duplication. Similar expression profiles and stress-responsive cis-acting elements (such as DRE, MYB, and ABRE motifs found in approximately 70% of chr6 GST promoters; **Supplementary Table S5**) are often shared by genes that reside in these clusters. This suggests coordinated transcriptional regulation and possible functional redundancy under abiotic stress, a pattern that is frequently seen in plant GST families. Despite significant phylogenetic distance, 14 conserved orthologous GST pairs were found between *Solanum lycopersicum* and *Crocus sativus* (**Figure 5B**), indicating the evolutionary conservation of this gene family across monocots and eudicots. The lack of GSTs on chr3 may represent lineage-specific genomic rearrangement or functional replacement by other detoxifying gene families.

### 3.6 GST proteins subcellular localization

Subcellular localization study by WolfPsort demonstrated varied distributions of GST proteins throughout cellular compartments, indicating their complex involvement in saffron’s physiology (**Supplementary Table S5**). The majority of GSTs, such as *CsGST1*, *CsGST11*, *CsGST24*, and *CsGST25*, are specified to reside in the cytoplasm, where they presumably detoxify reactive chemicals produced during secondary metabolite formation, hence preserving redox equilibrium in the stigma. Proteins like *CsGST17* and *CsGST18* are anticipated to reside in the nucleus and mitochondria, indicating potential functions in gene regulation or mitochondrial stress responses, which are essential during saffron’s energy-demanding flowering phase. Moreover, *CsGST16* and *CsGST7* are anticipated to localize in the chloroplast, thereby safeguarding photosynthetic tissues from reactive oxygen species during elevated light or drought stress, hence indirectly facilitating energy requirements for apocarotenoid formation. These localization patterns underscore the importance of GSTs to cellular integrity across many compartments, hence facilitating saffron’s capacity to synthesize high-quality secondary metabolites under environmental stress.

### 3.7 Physicochemical characteristics of GST proteins

The physicochemical properties of GST proteins further underscore their functional diversity (**Supplementary Table S6**). Molecular weights ranged from 20.49 to 68.49 kDa, and isoelectric points (pI) varied between 5.33 and 7.74, indicating a predominance of neutral to moderately acidic proteins. Grand Average of Hydropathy (GRAVY) scores, ranging from -0.281 to 0.143, confirm that most GSTs are hydrophilic, facilitating their enzymatic activity in aqueous cellular environments. The predominance of cytoplasmic GSTs, with some exhibiting dual localization in chloroplasts, nuclei, or mitochondria, aligns with their roles in detoxification and stress response. The hydrophilic nature and structural diversity of GSTs likely enable interactions with diverse substrates, including precursors or byproducts of crocin and safranal synthesis. For instance, chloroplast-localized GSTs may protect photosynthetic tissues, ensuring sufficient energy for secondary metabolism, while nuclear and mitochondrial GSTs stabilize cellular processes during stress, supporting saffron’s reproductive and metabolic demands.

### 3.8 Hormone-responsive element distribution in promoter regions

**Figure 6:**
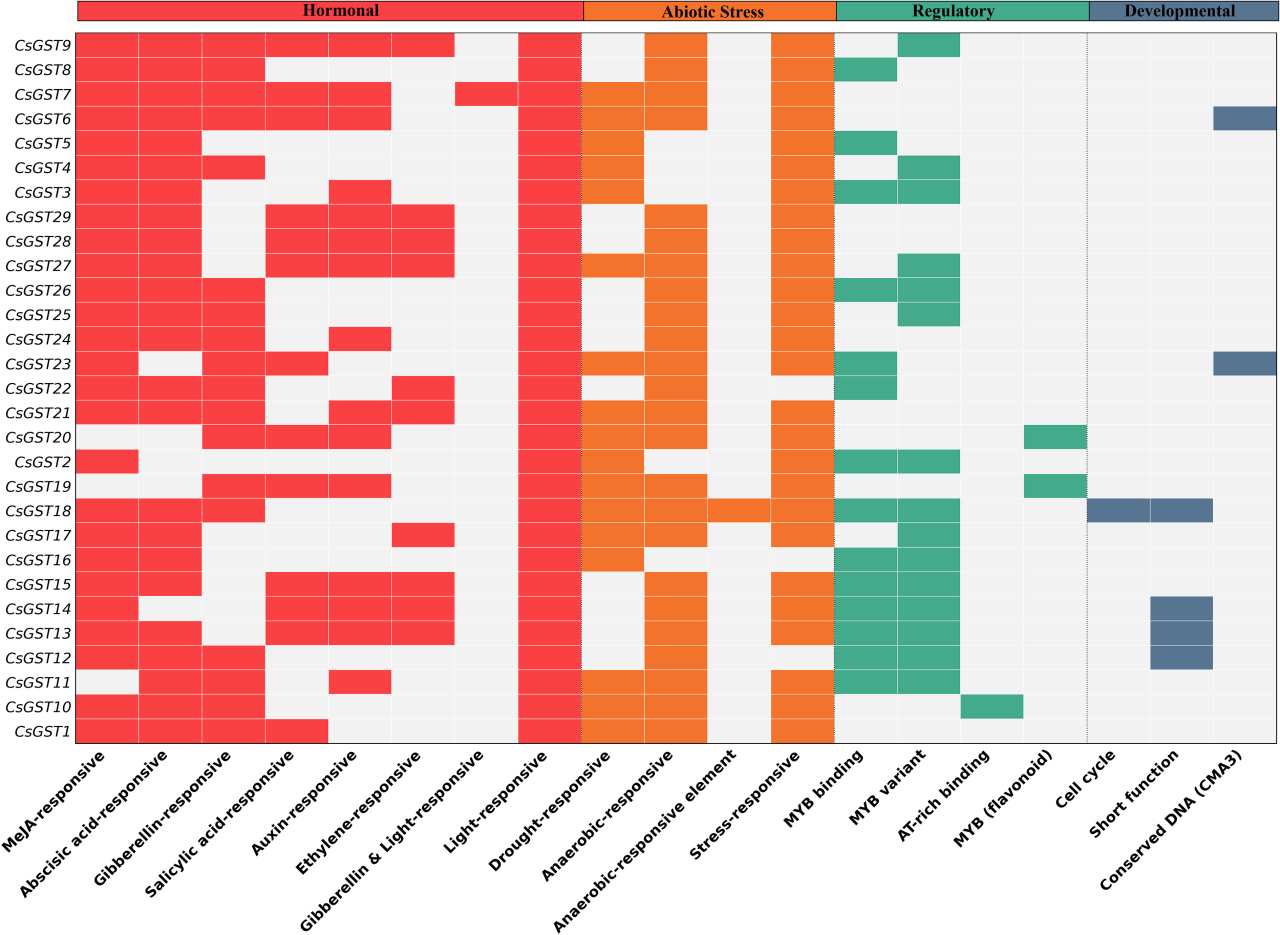
Distribution of cis-acting regulatory elements inside the 2000 bp upstream promoter regions of 29 C*. sativus* GST genes (*CsGST1* to *CsGST29*), as discovered by PlantCARE. Hormonal, Abiotic Stress, Regulatory, and Developmental categories are used to categorize functional motifs (section headers are placed above columns). The presence (colored) or absence (grey) of the motif is indicated by colored blocks, emphasizing the combinatorial nature of the GST gene family’s regulation in response to stress and hormones.

Analysis of the promoters of 29 C*. sativus* GST genes (*CsGST1* to *CsGST29*) identified 844 cis-acting regulatory elements in the 2000 bp upstream regions, classified as hormone-responsive, stress-responsive, light-responsive, and other regulatory motifs (**Supplementary Table S7**). Putative hormone-responsive elements were prevalent, comprising 236 MeJA-responsive motifs (118 CGTCA-motifs and 118 TGACG-motifs) identified across 29 genes, 120 ABRE motifs (ABA-responsive) in 27 genes, and 34 gibberellin-responsive motifs (6 GARE-motifs, 10 P-boxes, 18 TATC-boxes) in 20 genes, in addition to 29 salicylic acid-responsive elements (2 SARE, 27 TCA-elements), 17 auxin-responsive elements (GC-motif), and 20 ethylene-responsive elements (4 AACA-motifs, 16 GCN4-motifs), culminating in a total of 456 hormone-related motifs. Stress responsive elements included 76 anaerobic induction motifs (75 ARE, 1 TGA-box) across 25 genes, 26 drought-related MYB binding sites (MBS) in 18 genes, and 54 defense/stress-responsive motifs (44 LTR, 10 TC-rich repeats) in 24 genes, amounting to a total of 156 occurrences. Light-responsive elements were prevalent, including 364 motifs, including 92 G-box, 64 Box 4, 71 GT1-motif, and 38 TCCC-motif occurrences, identified in 29 genes, indicating possible light-mediated regulation. Additional regulatory components included 52 MYB binding sites (22 MRE, 2 MBSI, 28 CCAAT-box variations), 1 AT-rich region, 1 cell cycle MSA-like motif, and 2 3-AF3 binding sites (CMA3), amounting to a total of 68 occurrences. The findings underscore the potential regulation of *CsGST* genes by MeJA, ABA, and gibberellin (**Figure 6**), which are essential for biotic and abiotic stress responses in *C. sativus*, particularly in the production of secondary metabolites (e.g., crocin, safranal), while stress- and light-responsive motifs indicate a wider capacity for environmental adaptation.

### 3.9 Structural and interaction analysis of *CsGST* proteins

**Figure 7:**
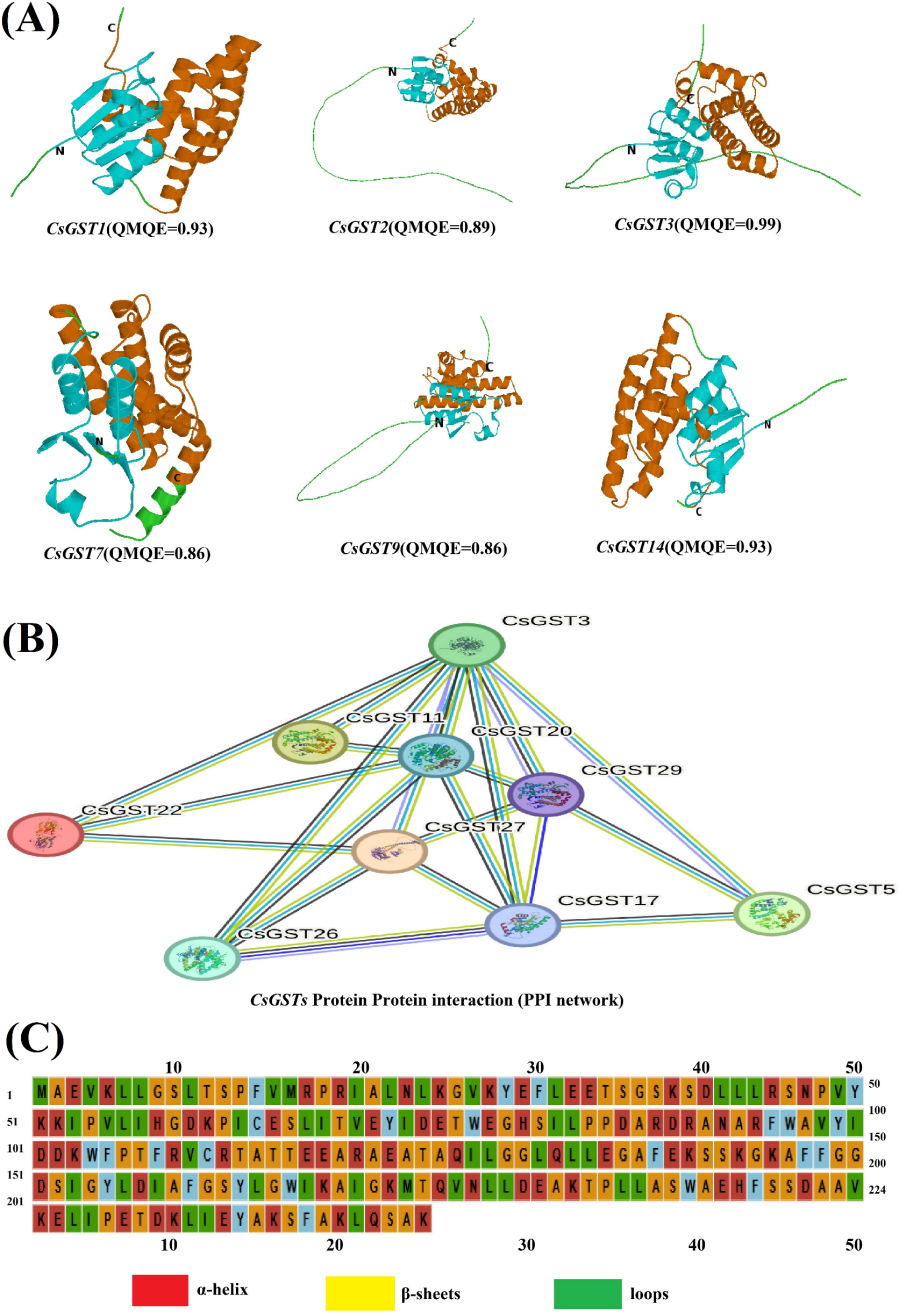
Analysis of the structure and interactions of *CsGST* proteins. **(A)** Ribbon diagrams of *CsGST1* (QMEAN = -0.93), *CsGST2* (QMEAN = -0.89), *CsGST3* (QMEAN = -0.89), *CsGST7* (QMEAN = -0.86), *CsGST9* (QMEAN = -0.86), and *CsGST14* (QMEAN = -0.93) illustrate the N-terminal (cyan) and C-terminal (orange) domains, with α-helices (red) and β-sheets (yellow) annotated. **(B)** The protein-protein interaction (PPI) network of the *CsGST* family. **(C)** Sequence alignment of selected *CsGST* proteins, illustrating conserved α-helices (red), β-sheets (yellow), and loops (green) according to PSIPRED and Alpha-fold predictions.

The QMEAN assessment of SWISS-MODEL-generated models for *CsGST1*, *CsGST2*, *CsGST3*, *CsGST7*, *CsGST9*, and *CsGST14* demonstrated substantial structural reliability, with QMEAN scores varying from -0.93 (*CsGST1*, *CsGST14*) to -0.86 (*CsGST7*, *CsGST9*). Ribbon diagrams (**Figure 7A**) demonstrated conserved GST characteristics: a thioredoxin-like N-terminal domain (residues 10-91) comprising β-strands and α-helices that constitute the GSH-binding site (G-site, e.g., Ser15, Arg20 in *CsGST1*) for substrate specificity, and a predominantly α-helical C-terminal domain (residues 96-221) containing the hydrophobic H-site for xenobiotic binding. PSIPRED and AlphaFold predictions were closely correlated, validating the presence of α-helices (red) and β-sheets (yellow) essential for catalytic activity. The protein-protein interaction (PPI) network (**Figure 7B**) showed *CsGST3* as a prominent hub, interacting with *CsGST11*, *CsGST17*, *CsGST20*, and *CsGST29*, indicating its pivotal involvement in orchestrating hormone-mediated stress response pathways (e.g., MeJA, ABA-driven detoxification). Sequence alignments (**Figure 7C**) emphasized conserved secondary structures α-helices (red), β-sheets (yellow), loops (green) that support the functional roles of *CsGSTs* in saffron’s stress resilience.

### 3.10 Hormone-mediated regulation of GST gene expression in *C. sativus*

RNA-seq analysis of datasets PRJNA400472 and PRJNA976833 demonstrated considerable differential expression of GST genes in *C. sativus*, indicating their regulation by MeJA, ABA, and GA during stigma formation, tissue specific stress responses, and bud dormancy (**Figure 1**). Genes with false discovery rate (FDR) < 0.05 (Benjamini-Hochberg adjusted p-value from DESeq2) were considered differentially expressed, *CsGST1* (logFC = 0.423, FDR = 0.038), *CsGST13* (logFC = 0.116, FDR = 0.019), and *CsGST23* (logFC = 0.270, FDR = 0.021) exhibited modest but statistically significant upregulation during stigma maturation (Stigma_RED vs Stigma_0_DAY, **Figure 1**). The significant upregulation of *CsGST1* corresponds with MeJA- and GA-responsive regions in its promoter, possibly aiding in the detoxification of reactive oxygen species during crocin production in the RED stigma stage, which is essential for saffron quality. The overexpression of *CsGST23* is associated with ABA-responsive components, reinforcing ABA’s function in late stigma formation under dehydration stress, hence improving reproductive success. Heatmap B (PRJNA400472) shows the expression of the *CsGST* gene in stigma tissue on Days R1, R2, and R3 during stress treatment. *CsGST23* had elevated expression on Day R1, indicating an early MeJA/ABA-mediated function in stress response, while *CsGST28* displayed high expression on Day R3, signifying a prolonged GA-driven response. *CsGST25* and *CsGST19* exhibited moderate expression over all days, indicating persistent stress responses, whereas genes such as *CsGST1*, *CsGST5*, and *CsGST2* had low or negligible expression, suggesting little participation in stigma stress responses under these circumstances. In tissue-specific comparisons (PRJNA976833, Apical_Bud_Nov vs. Leaf_Nov), *CsGST9*, *CsGST21*, *CsGST24*, *CsGST25*, *CsGST26*, and *CsGST27* exhibited substantial upregulation in apical buds (logFC = 2.190, 3.248, 2.556, 1.047, 1.187, 3.554; FDR < 0.05). *CsGST27* demonstrated the most significant upregulation (logFC = 3.554), closely associated with GA-responsive components, highlighting GA’s involvement in modulating bud dormancy in November, a crucial adaptation for winter survival. The increased expression of *CsGST9* indicates a stress response mediated by MeJA/ABA in buds, perhaps safeguarding against oxidative damage generated by cold. In contrast, *CsGST13* and *CsGST14* exhibited substantial downregulation in buds (logFC = -2.448, -3.559; FDR < 10^-5^), suggesting less MeJA/ABA signaling relative to leaves, where these hormones might facilitate photosynthetic stress responses. Heatmap A (PRJNA976833) showed *CsGST* expression in diverse tissues (daughter corm, leaf, apical bud, etc.) under varying experimental circumstances. *CsGST18*, *CsGST25*, and *CsGST17* exhibited elevated expression in leaves and daughter corms, indicating significant involvement in stress reactions, perhaps mediated by MeJA/ABA. *CsGST28* demonstrated moderate to high expression in all tissues, suggesting a widespread GA-mediated function in stress responses. *CsGST5* and *CsGST6* exhibited moderate expression in leaf and daughter corm, but *CsGST4*, *CsGST9*, and *CsGST10* had low or negligible expression in most tissues, suggesting restricted participation in the evaluated circumstances. To confirm hormone-mediated regulation, we examined the distribution of MeJA-, ABA-, and GA-responsive regions in the promoters of differentially expressed GST genes, classified into UR-S, DR-S, UR-L/C/B, and DR-L/C/B groups. A Venn diagram (**Figure 1D**), created using Venny 2.1, illustrated the intersection of hormone-responsive parts. Five genes (*CsGST1*, *CsGST19*, *CsGST23*, *CsGST28*, *CsGST29*) exhibited MeJA/GA-responsive components in both UR-S and UR-L/C/B, corroborating their involvement in oxidative stress responses. Three genes (*CsGST12*, *CsGST13*, *CsGST22*) exhibited common components across UR-S, UR-L/C/B, and DR-S, suggesting intricate regulation. Fifteen genes (*CsGST2, CsGST3, CsGST6, CsGST7, CsGST9*, *CsGST14*, *CsGST15*, *CsGST16*, *CsGST17*, *CsGST18, CsGST20, CsGST24, CsGST25, CsGST26, CsGST27*) exhibited GA-responsive components throughout UR-L/C/B and DR-S, underscoring GA’s significance in bud dormancy. Three genes (*CsGST8, CsGST4, CsGST5*) exhibited ABA-responsive elements across UR-S, DR-L/C/B, and DR-S, while *CsGST10*, *CsGST11*, and *CsGST21* displayed elements across DR-L/C/B and DR-S, underscoring tissue-specific ABA regulation. GO enrichment analysis was conducted on 18 substantially differentially expressed *CsGST* genes (FDR < 0.05) to clarify their functional functions. Significant GO keywords (FDR p-value < 0.05) were found using eggNOG-mapper annotations and a UniProt background for cucumber (*Cucumis sativus*). Despite being a monocot, *Crocus sativus* has less thorough Gene Ontology annotations than well-annotated eudicots. In order to optimize statistical power and word coverage, *Cucumis sativus* (cucumber), which now provides the most comprehensive and high-quality plant GO annotation collection among fully sequenced species, was used as the background reference. The biological significance of the enrichment data is unaffected by this selection since GO keywords linked to stress and hormones are substantially conserved across angiosperms. The filtration of hormone and stress related terminology indicated an enrichment in processes including cellular response to stress, response to abscisic acid, and gibberellin mediated signaling pathway, thereby affirming the roles of MeJA, ABA, and GA in stress responses and developmental regulation (**Figure 1C**). The data jointly affirm that MeJA, ABA, and GA regulate GST gene expression in *C. sativus*, affecting stigma formation, stress responses, and bud dormancy.

## 4 Discussion

Members of the GST gene family has been reported to play a crucial role in the stress tolerance in several plant species including *O. sativa*, *G. max*, *Z. mays*, pepper, and tomato (Islam et al., 2019). Despite the potential role of GSTs in important physiological and defense processes, no comprehensive information is available in *C. sativus* on the GST family. GSTs are crucial for hormone-mediated stress resilience and developmental control, allowing saffron to flourish in the demanding Mediterranean climate and enhancing its global agricultural importance as a high-value crop. This study clarifies the functional diversification and evolutionary dynamics of the *CsGST* gene family, offering biological insights to improve crop resilience and production. Through the integration of phylogenetic analysis, RNA-seq expression patterns, and genomic distribution, we elucidate the role of *CsGSTs* in orchestrating adaptive responses, providing a basis for targeted enhancements in saffron farming. Phylogenetic study categorizes *CsGSTs* into the Phi, Tau, Theta, and Lambda subclasses, each adapted to certain ecological conditions. In the present study, we identified 29 *CsGSTs* distributed across the genome. Genomic distribution revealed that the *CsGSTs* have an uneven distribution across the genome.

Phylogenetic analysis classified the majority of the *CsGSTs* into Phi clade (13 genes), followed by Tau clade (10 genes). The remaining genes were grouped into other clades (theta and lambda). The majority of the *CsGSTs* are grouped into 2 clades (Phi and Tau). Phi-class *CsGSTs* presumably neutralize reactive oxygen species (ROS) and xenobiotics in stigma tissues, hence safeguarding reproductive success essential for saffron’s economic significance. Tau-class genes, conversely, augment resistance to abiotic stresses including drought and heat, common in saffron’s indigenous habitat. This functional specialization corresponds with observations in *Oryza sativa*, where Tau GSTs (e.g., *OsGSTU30*) provide drought and heavy metal tolerance, and in *Arabidopsis thaliana*, where Phi GSTs (e.g., *AtGSTF8*) alleviate biotic stress (Srivastava et al., 2019). The Theta and Lambda subtypes, albeit less prevalent, probably aid in reducing oxidative stress and facilitating specialized metabolic activities, hence enhancing saffron’s adaptive capabilities. The evolutionary divergence indicates that *CsGSTs* have experienced sub-functionalization, enabling saffron to respond to many stresses despite its triploid sterility, a limitation that restricts genetic recombination. The chromosomal distribution of *CsGSTs*, segmental duplications on Chromosomes 1, 5, and 6, is likely attributable to tandem duplications, a process similarly found in Secale cereale, where GST gene clusters augment stress resilience (Shi et al., 2024). These genomic hotspots may enable synchronized expression with neighboring stress-related genes, such as cytochrome P450s or heat shock proteins, which are frequently co-localized in stress-responsive areas of plant genomes (Gallé et al., 2009). In saffron, such interactions may enhance stress response capabilities, compensating for the absence of sexual reproduction. For example, cytochrome P450s, recognized for detoxifying xenobiotics, may collaborate with Phi-class *CsGSTs* in stigma tissues, whereas heat shock proteins could augment the functions of Tau-class *CsGSTs* in abiotic stress tolerance. This notion necessitates additional examination via chromatin interaction studies or co-expression network analysis to clarify putative regulatory networks on these chromosomes, an essential step for comprehending saffron’s genomic adaptations.

RNA-seq revealed tissue-specific expression patterns that highlight the involvement of *CsGSTs* in stress adaption and development. *CsGST16* and *CsGST9* demonstrate increased expression in stigma tissues, alleviating oxidative stress during reproductive development, similar to the function of GSTs in *Lilium longiflorum* that safeguard floral tissues from reactive oxygen species. Likewise, the ABA-mediated expression of *CsGST23* in the late stigma enhances dehydration tolerance, which is essential for preserving stigma integrity in water-scarce environments. The GA-induced expression of *CsGST27* in apical buds promotes dormancy, allowing saffron to endure seasonal stressors. The expression patterns, influenced by MeJA, ABA, and light-responsive cis-regulatory elements, demonstrate stringent hormonal regulation, paralleling the GSTs of *Arabidopsis thaliana*, where MeJA and ABA signaling regulate stress responses (Sappl et al., 2004). These findings underscore the role of *CsGSTs* in integrating hormonal and environmental signals to equilibrate reproductive and vegetative growth within saffron’s resource-limited lifetime. The constant structural characteristics of *CsGSTs*, comprising thioredoxin-like N-terminal and helical C-terminal domains, facilitate effective glutathione conjugation, hence maintaining cellular metabolism during stress. The protein-protein interaction network, featuring *CsGST3* as a major hub, indicates coordinated communication between MeJA and ABA pathways, perhaps involving interactions with stress-related proteins such as peroxidases, as documented in (Islam et al., 2019). These interactions augment saffron’s capacity to sustain metabolic homeostasis amid environmental variations. The dependence on cucumber-derived GO annotations may neglect saffron-specific functions, especially considering its distinct triploid genome. The omission of distal promoter elements beyond 2000 bp constrains the comprehension of long-range regulation mechanisms. Subsequent investigations may confirm the detoxifying functions of *CsGST16* and *CsGST9* by biochemical tests, examine the relationships of *CsGST3* using co-immunoprecipitation, or utilize CRISPR-based editing to augment stress tolerance. Comparative genomics with other bulbous plants may elucidate the evolutionary adaptations of *CsGSTs*. *CsGSTs* serve as molecular targets to enhance saffron’s flexibility by mediating stigma protection, corm resilience, and metabolic stability, thereby satisfying the global demand for this high-value crop and promoting sustainable agriculture. The *CsGST* gene family of saffron exhibits unique species-specific traits that are shaped by its triploid sterility and adaptation to arid, high-light environments: strong enrichment of MeJA-, ABA-, and light-responsive cis-elements in stigma and bud-specific members; dominance of Phi and Tau classes; lack of DHAR and TCHQD subfamilies; and prominent clustering on chromosomes 1, 5, and 6. These characteristics most likely developed to ensure corm survival during seasonal drought and temperature fluctuations and to shield the economically important stigma from oxidative damage. Promoter analysis was restricted to 2-kb upstream areas, expression profiles were obtained from publicly available RNA-seq datasets rather than controlled treatments, and functional designations are still predictive since this work is fully in-silico. These limitations are typical for non-model species and do not affect the accuracy of the expression patterns or genome inventory. The following specific hypotheses should be tested in future research: (i) *CsGST9*, *CsGST16*, and *CsGST23* directly protect crocin biosynthesis from oxidative stress (as confirmed by VIGS or overexpression with apocarotenoid quantification under drought/MeJA); (ii) *CsGST14* and *CsGST27* control GA-mediated dormancy and cold tolerance in buds (as tested by qRT-PCR and enzyme assays under GA/low temperature); and (iii) *CsGST3* acts as a central hub coordinating MeJA-ABA signaling (confirmed by yeast two-hybrid or co-immunoprecipitation). In addition to supporting breeding for increased stress tolerance and spice quality, this focused validation will move saffron GST research from prediction to function.

## 5 Conclusions

This comprehensive study focused on the identification, annotation, and functional analysis of glutathione S-transferase (GST) genes in *C. sativus*. Twenty-nine GST genes were identified through genome-wide searches and categorized into distinct subfamilies based on conserved domains, motif characteristics, and evolutionary relationships. Chromosomal distribution analysis revealed clustering of GST genes, indicating gene duplication events. Ka/Ks analysis suggested that purifying selection is the primary evolutionary driver, with some evidence of relaxed purifying selection pointing to functional diversity. Promoter analysis uncovered hormone-responsive cis-elements, including MEJA, ABRE, and GARE, highlighting the role of GST genes in hormone-mediated stress responses. Predictions regarding subcellular localization showed that GST proteins are predominantly localized in the cytoplasm, with occasional presence in the nucleus, vacuole, or chloroplast, emphasizing their versatile functions. The structural characteristics of a GST protein were analyzed using the Swiss-Model platform, revealing the N-terminal and C-terminal domains responsible for substrate binding and catalytic activity. The reliability of the model was confirmed through ligand interaction analysis, which identified key residues potentially involved in functional connections. These results augment our comprehension of the structure, evolution, and function of GST genes in *C. sativus*, establishing a basis for future investigations into GST-mediated stress tolerance, plant development, and the enhancement of crop resilience and secondary metabolite production.

## Data Availability

The original contributions presented in the study are included in the article/**Supplementary Material**. Further inquiries can be directed to the corresponding authors.

## Supplementary material

Supplementary Table 1 Genomic information of *Crocus sativus* GST genes.

Supplementary Table 2 Glutathione S-transferase genes in *Crocus sativus*.

Supplementary Table 3 Motif sequences, logo and widths.

Supplementary Table 4 Evolutionary analysis of GST gene family in *Crocus sativus*.

Supplementary Table 5 Subcellular distribution of *CsGST* genes.

Supplementary Table 6 Overview of *CsGST* proteins.

Supplementary Table 7 Cis-acting regulatory elements in *CsGST* promoters.
