# Supplementary material for: Genome-wide identification and in silico analysis of GSTs reveals hormone mediated stress response in saffron
Source: Front Plant Sci. 2026 Jan 9;16:1676384. doi: 10.3389/fpls.2025.1676384 (PMC12827570; doi:10.3389/fpls.2025.1676384)
Supplement: Supplementary Table 5 — Subcellular distribution of CsGST genes. [file Table5.docx]

**Table S5**

**Subcellular Distribution of CsGST Genes**

| **Gene Name** | **cyto** | **cyk** | **chlo** | **extr** | **nucl** | **pero** | **plas** | **golg** | **mito** | **vacu** | nucl_plas |
| --- | --- | --- | --- | --- | --- | --- | --- | --- | --- | --- | --- |
| *CsGST-1* | *5* |  | *2* |  | *2* |  | *3* |  | *2* |  | *3.5* |
| *CsGST-2* | *5* |  | *2* |  | *2* |  | *2* |  | *4* |  | *3* |
| *CsGST-3* | *5* |  | *1* |  | *2* |  | *2* |  |  |  | *3* |
| *CsGST-4* |  |  | *10* | *2* | *1* |  |  |  | *1* |  |  |
| *CsGST-5* |  |  | *11* | *2* | *1* |  |  |  |  |  |  |
| *CsGST-6* | *1* |  | *12* | *1* |  |  |  |  |  |  |  |
| *CsGST-7* | *1* |  | *10* | *3* |  |  |  |  |  |  |  |
| *CsGST-8* | *1* | *11* |  | *1* |  |  | *1* |  |  |  |  |
| *CsGST-9* |  |  | *12* | *1* | *1* |  |  |  |  |  |  |
| *CsGST-10* | *9* | *1* | *1* |  |  | *2* |  | *1* | *1* |  |  |
| *CsGST-11* | *10* |  | *2* |  |  | *1* |  | *1* |  |  |  |
| *CsGST-12* | *8* |  |  |  | *4* | *1* | *1* |  |  |  |  |
| *CsGST-13* | *11* |  | *3* |  |  |  |  |  |  |  |  |
| *CsGST-14* | *12* |  | *2* |  |  |  |  |  |  |  |  |
| *CsGST-15* | *11* |  | *3* |  |  |  |  |  |  |  |  |
| *CsGST-16* |  |  | *12* |  | *1* |  |  |  | *1* |  |  |
| *CsGST-17* |  |  | *11* |  |  | *1* |  |  | *2* |  |  |
| *CsGST-18* | *2* |  | *5* |  | *1* |  |  |  | *6* |  |  |
| *CsGST-19* | *3.5* |  | *1* |  |  | *8* |  | *1* |  |  |  |
| *CsGST-20* | *3.5* |  | *1* |  |  | *8* |  | *1* |  |  |  |
| *CsGST-21* | *2* |  | *6* | *3* |  | *1* |  |  |  |  |  |
| *CsGST-22* | *9* |  |  |  | *3* | *1* |  |  | *1* |  |  |
| *CsGST-23* | *2* |  | *11* | *1* |  |  |  |  |  |  |  |
| *CsGST-24* | *9* |  | *3* |  | *1* |  |  | *1* |  |  |  |
| *CsGST-25* | *11* |  | *2* |  |  |  |  | *1* |  |  |  |
| *CsGST-26* | *10.5* | *1* | *1* |  |  |  |  | *1* |  |  |  |
| *CsGST-27* | *2.5* |  | *2* |  | *1.5* | *7* |  | *1* |  |  |  |
| *CsGST-28* | *1* |  | *12* | *1* |  |  |  |  |  |  |  |
| *CsGST-29* | *1* |  | *12* | *1* |  |  |  |  |  |  |  |
